# Can early surgery improve the outcome of patients with meconium peritonitis? A single-center experience over 16 years

**DOI:** 10.1186/s12887-019-1844-5

**Published:** 2019-12-03

**Authors:** Yi Jiang, Weihua Pan, Wenjie Wu, Weipeng Wang, Suna Sun, Jun Wang

**Affiliations:** Department of Pediatric Surgery, Xinhua hospital; Shanghai Jiao Tong University School of Medicine, No. 1665, Kongjiang Road, Shanghai, 200092 China

**Keywords:** Meconium peritonitis, Early operation, Survival rate, Premature, Risk factor

## Abstract

**Background:**

In the last century, meconium peritonitis(MP)was once a highly fatal gastrointestinal. disease With the development of fetal radiological technology, abnormal signs, such as pseudocysts, can. be detected during the fetal period so that more patients can be diagnosed prenatally and receive surgery. in the early stage of life. The survival rate of MP has increased up to 80% in recent years. According to. a review of the treatment and outcomes of patients diagnosed with MP, we evaluated the influence of. early operation on survival rate and discussed the risk factors of prognosis.

**Methods:**

We collected 79 cases of patients diagnosed with MP who were treated in our department. from October 2001 to December 2017. They were divided into 2 groups. Patients in group A were born. in our hospital. Patients in group B were born in a local hospital with suspicion of MP and then transferred. to our department.

**Results:**

The birth weight (BW) and gestational age (GA) of patients were higher in group A than in. group B. There was no significant difference in the proportion of premature and low birth weight (LBW). patients between the two groups (*p =* 0.422, *p =* 0.970). Their age at the time of surgery was younger in. group A than in group B (1.4 ± 2.0 vs. 6.9 ± 14.9, *p* < 0.001). The overall survival rate of group A was higher. than that of group B (95.0% vs. 79.5%, *p =* 0.038). The prognosis of premature patients was worse than. that of full-term infants for both groups (*p =* 0.012).

**Conclusions:**

Prematurity is a significant risk factor related to death for MP patients. The survival rate. of MP patients can be improved by early operation during the neonatal period.

## Background

Meconium peritonitis (MP) is a rare gastrointestinal disease, with an incidence between 1/30000 and 1/35000 [1–4]. When perinatal intestinal perforation occurs, usually caused by intestinal atresia, stenosis or malrotation, meconium enters the abdominal cavity and causes sterile chemical peritonitis. With the development of radiological technology, abnormal signs such as fetal ascites, bowel dilatation, intraabdominal calcification and pseudocyst, can be detected during fetal period by ultrasound (US) and magnetic resonance imaging (MRI), so that more can be diagnosed prenatally and receive operation in early stage of life. The survival rate of MP has been increased up to 80% recent years [2, 5–7].

Several single- and multicenter studies have proposed that fetal patients diagnosed with MP should be delivered in a tertiary center to undergo early operation to improve the survival rate and prognosis [2, 8–11]. Early operation leads to earlier repair and restoration, providing additional time for the bowel to recover and develop in the neonatal period. Animal experiments showed that lack of a microbiome could impair adrenal catecholamine, implying that the poor health status of neonates might be caused by immature of intestinal function [12]. However, the choice of surgical method remains controversial. The choice of procedures, including primary anastomosis and enterostomy, usually depends on the condition of bowel and the severity of intraabdominal infection found during operation. Here, we describe our experience in the treatment and management of patients with MP and analyze the effects of early operation to provide some potential risk factors for MP.

## Methods

From October 2001 to December 2017, 82 patients (45 males and 34 females) with MP were admitted to our department. Three of them who did not accept surgery were excluded. A total of 79 patients were ultimately included in the retrospective study. We collected data, including sex, gestational age (GA), delivery mode, birth weight (BW), antenatal diagnosis, white blood cell count, C-reaction protein (CRP) and albumin (ALB) levels before operation, type of operation, length of remaining bowel after operation, time of parenteral and enteral nutrition administration, postoperative complications, outcomes and hospital length of stay (LOS).

We divided the 79 patients into two groups to evaluate the influence of early operation on survival rate. Forty children born in our hospital underwent early operation, mostly in the first 24 h after birth, and were included in group A. Thirty-nine patients who were transferred to our department of pediatric surgery were included in group B.

The SPSS v.23.0 was used for statistical analysis. The Shapiro-Wilk test was used to test the normal distribution of the variables. The Mann Whitney test was used to compare variables with abnormal distributions. Qualitative variables between groups were compared by the Chi square test. A *p*-value of less than 0.05 was considered significant.

## Results

Overall, the GA was between 28 and 41^+ 4^ weeks (M = 37.2 ± 2.3), and the BW ranged from 1330 g to 4280 g (M = 3162.1 ± 532.3). The average age at the time of o surgery was 4.1 ± 10.8 days after birth (M = 1, range 0 to 83). The overall survival rate was 87.3%. A total of 69 patients were cured and discharged from the hospital without respiratory support or parenteral nutrition. The average hospitalization time was 23.9 ± 13.6 days (M = 20, range 12 to 84). The average duration of parenteral nutrition utilization was 16.5 ± 6.7 days (M = 15, range 8 to 40). Four patients died of severe infection, and two died of respiratory failure. Four families discontinued the treatments due to multimalformation of the child and financial problems.

Among these patients, intraabdominal calcification was found in 6 patients and intraabdominal pseudocysts were found in 10 patients before exploratory laparotomy. The remaining patients underwent exploratory laparotomy because of abdominal distention and tenderness. During the surgery, pseudocysts were found in another 25 patients. Complex intestinal adhesion with diffuse turbid ascites in the abdominal cavity or purulent adhesion to the abdominal wall were found in the remaining 38 patients, which verified the diagnosis of meconium peritonitis. The methods of surgery included peritoneal drainage (1 case, 1.3%), enterolysis (1 case, 1.3%), intestinal resection and anastomosis (34 cases, 43.0%) and enterostomy (43 patients, 54.5%). The average length of remaining bowel of all the patients was 105.3 ± 42.3 cm. Postoperative complications occurred in 9 of 77 patients who received anastomosis and enterostomy, with a morbidity of 11.7%. The morbidity of surgical complications was 8.8% for primary anastomosis (one intestinal fistula and two intestinal obstructions) and 9.3% for enterostomy (one wound infection, one stoma prolapse and four intestinal obstructions), while the survival rate were 88.2 and 86.0% (*p* > 0.05), respectively (Table 1).

**Table 1:** Clinical indicators of different methods of surgery

Procedures	Enterostomy (*n* = 43)	Primary anastomosis (*n* = 34)	*p* value
Length of remaining bowel (cm)	98.5 ± 33.9 (M = 99.5)	112.8 ± 49.7 (110.0)	0.215^a^
Length of parenteral nutrition (d)	16.3 ± 6.2 (M = 15)	16.6 ± 7.2 (M = 14)	0.843^a^
Start time of enteral nutrition (d)	8.7 ± 4.1 (M = 8.0)	9.4 ± 6.0 (M = 6.0)	0.967^a^
LOS (d)	22.0 ± 12.3 (M = 21.0)	21.8 ± 15.8 (M = 18.0)	0.098^a^
Complication	6 (14.0%)	3 (8.8%)	> 0.05^a^
Survival	37 (86.0%)	30 (88.2%)	> 0.05^a^

^a^Mann Whitney Test: Length of remaining bowel: U = 327.5, Z = -1.240

PN: U = 199.5, Z = -0.198; EN: U = 194.0, Z = -0.041; LOS: U = 570.0, Z = -1.656; Complication: χ2=, *p* > 0.05; Survival rate: χ2=, *p* > 0.05.

Among the 56 of 79 patients (70.9%) who were prenatally diagnosed with MP, a survival rate of 87.50% was observed. The most frequent findings of fetal US and MRI were bowel dilatation (44, 78.6%), followed by polyhydramnios (28, 50%), ascites (15, 26.8%), echogenic bowel (6, 10.7%), intraabdominal calcification (6, 7.1%), intraabdominal pseudocyst (4, 7.1%) and oligohydramnios (1, 1.8%). The average age at the time of surgery operation of the 79 patients was 4.1 ± 10.8 days, and the average LOS was 22.2 days. For those fetuses who were diagnosed with MP, fetal US examination was conducted to monitor the growth of the fetus once per week during the third trimester of pregnancy. Selective delivery was be conducted by obstetricians if a new abnormal sign was found or if the existing signals worsened, although the desired GA was more than 37 weeks. Reinforcement of nutrition for gravidas, including diets with high-quality protein and glycemic control, was formulated by nutritionists. As a result, 38 of these patients who received the mentioned treatment were born in our tertiary center with an average GA of 37.8 ± 1.8 weeks, which is better than the GA of 36.4 ± 2.2 weeks for the other 18 patients (*p* = 0.024). Among all the patients, 49 were delivered via cesarean section, and 29 were vaginally delivered (one abandoned baby was not included due to the absence of a recorded delivery mode). There was no difference in the survival rate between patients delivered by the two different modes (87.8% vs. 86.2%, χ2 = 0.0233, *p* > 0.05).

### Statistics of demographics and treatment data between the two groups

Thirty-one patients (77.5%) in group A underwent surgery in the first day after birth, while only 13 patients (33.3%) in group B underwent surgery within the first day (one abandoned baby was not included due to the absence of a recorded age). Patients in Group B were diagnosed with ileus or meconium peritonitis first in a local hospital and then transferred to our center at 4.4 ± 12.0 days (range 0 to 71, median = 1) after birth. Then, some examinations were required before the anesthesia and surgery, including blood coagulation testing, chest radiography, echocardiography, etc. The average age of patients at the time of surgery in group A was 1.4 ± 2.0 days (M = 1, range 0, 12), which was significantly younger than that in group B (1.4 ± 2.0 days vs. 6.9 ± 14.9 days, *p* < 0.001) (Table 2). The survival rate of group A was also higher than that of group B (95.0% vs. 79.5%, *p* = 0.038). Similar to the difference in the rate of fetal diagnosis between the two groups (95.0% vs. 46.2%, p < 0.001), the gestational age and birth weight of neonates in group A were both higher than those in group B (37.8 ± 1.8 vs. 36.4 ± 2.7, *p* = 0.015, 3288.9 ± 521.6 vs. 3025.1 ± 516.1, *p* = 0.029, respectively). However, there was no difference in the proportion of premature or low birth weight (LBW) patients between the two groups (*p* = 0.422, *p* = 0.970). To assess the general conditions of the patients, we analyzed indexes of hematological and biochemical indicators and markers of inflammation. The C-reaction protein (CRP) value was measured in 35 children in group A, 9 of whom had a CRP value higher than 8 mg/L; ascites was collected and cultured for 29 patients, 7 of whom had positive culture results (24.1%). In group B, the CRP value was measured in 33 children, and 13 had elevated levels. Ascites bacterial culture was performed in 21 of 39 patients, and 8 were positive (38.1%). Although there was no difference between the two groups in the level of hemoglobin, blood platelets, albumin and white blood cell count (*p* = 0.912, *p* = 0.362, *p* = 0.391, and *p* = 0.166, respectively), the positive rates of CRP elevation and of ascites bacterial culture were lower in group A patients than in group B patients, although those difference were not statistically significant (25.7% vs. 39.4%, *p* = 0.228, 24.1% vs. 38.1%, *p* = 0.288, respectively).

**Table 2:** Characteristics and outcomes of patients in two groups

	Group A (*n* = 40)	Group B (*n* = 39)	*p* value
Male	24 (60.0%)	21 (53.8%)	0.581^#^
Preterm	11 (27.5%)	14 (35.9%)	0.422^#^
LBW	4 (10%)	4 (10.3%)	0.970^#^
Caesarean Section	26 (65.0%)	23 (59.0%)	0.304^#^
Fetal diagnosis	38 (95.0%)	18 (46.2%)	< 0.001^#^
Survival	38 (95.0%)	31 (79.5%)	0.038^#^
Age at operation (d)	1.4 ± 2.0	6.9 ± 14.9	< 0.001^a^
GA (w)	37.8 ± 1.8	36.4 ± 2.7	0.015^#^
BW (g)	3288.9 ± 521.6	3025.1 ± 516.1	0.029^#^
WBC (10^9/L)	14.7 ± 7.4	12.7 ± 5.7	0.166*
Hb (g/L)	150 ± 32	151 ± 28	0.912^#^
Plt (10^9/L)	219 ± 99	238 ± 88	0.362^#^
ALB (g/L)	32.4 ± 6.0	31.3 ± 4.7	0.391^#^
LOS (d)	23.1 ± 12.0	25.0 ± 15.4	0.904^a^

Abbreviations: *GA* gestational age; *BW* birth weight; *WBC* white blood cell; *Hb* hemoglobin; *Plt* blood platelet; *ALB* albumin level in serum; *LOS* length of stay in hospital. All the indicators of hematologic examination in the table were done before the operation

^#^ T-Test; ^a^Mann Whitney Test: Age at operation: U = 370.5, Z = -4.103; WBC: U = 605, Z = -1.386; LOS: U = 579.0, Z = -0.121; WBC: U = 499.5, Z = -2.461

### Statistics of demographics and treatment data in preterm and full-term patients

A total of 53 of 79 patients were full-term infants (67.1%), whose survival rate reached 94.3% (Table 3). In contrast, 25 preterm infants, whose GA was less than 37 weeks had a survival rate of only 72.0%. For preterm patients, the albumin value before surgery was much lower than that for full-term patients (28.9 ± 4.7 vs. 33.5 ± 5.0, *p* < 0.001). The average length of remaining bowel after surgery in preterm patients was shorter than that in full-term patients, although this difference was not significant (91.4 ± 40.3 vs. 112.8 ± 42.0, *p* = 0.055). Comparing the LOS, preterm patients needed more time in the hospital for treatment than full-term patients (28.6 ± 13.6 vs. 22.3 ± 13.4, *p* = 0.005). According to a logistic regression, there was a correlation between the outcome and premature delivery (*p* = 0.012). Among preterm infants, 8 had LBW, and only 5 survived. For low-birth weight patients, the survival rate was much lower than that for non-LBW patients, as expected (62.5% vs. 91.3%, *p =* 0.016).

**Table 3:** Characteristics of preterm and term infants

	Preterm infants	Term infants	*p* value
BW (g)	2713.1 ± 569.8	3365.5 ± 367.9	< 0.001#
ALB (g/L)	28.9 ± 4.7	33.5 ± 5.0	< 0.001#
Remaining bowel (cm)	91.4 ± 40.3	112.8 ± 42.0	0.055^a^
LOS (d)	28.6 ± 13.6	22.3 ± 13.4	0.005^a^
Survival	18 (72.0%)	50 (94.3%)	0.006#

Abbreviations: Remaining bowel, length of remaining bowel after operation

# T-Test; ^a^Mann Whitney Test, Remaining bowel: U = 255.5, Z = -1.917; LOS: U = 250, Z = -2.789

### Follow-up information

Thirty-five children were followed up from 39 days to 14 years (M = 3 years) in our clinics. One child was diagnosed with growth retardation, and the other 34 children grew well, according to the weight-for-age curve and height-for-age curve of the World Health Organization. The other 34 living patients returned to their local city and were lost to follow up.

## Discussion

With advances in medical imaging technology, most MP patients can be diagnosed antenatally by fetal US or MRI, which contributes to the early application of surgery for neonates. Shyu M et al. [8] demonstrated that the prognosis of MP patients with ileus can be improved by early operation. Keiichi Uchida et al. [10] also verified that early surgery is an effective way to reduce intra-abdominal and systemic inflammation, which helps to enhance the outcome of severely affected patients. In our study, the much higher prenatal diagnosis rate and survival rate in group A presented significant positive effects on the prognosis due to early application of surgery, even if the rate of preterm and low birth weight patients were similar between the two groups. MP is usually caused by gastrointestinal malformation such as intestinal atresia, stenosis and malrotation [1, 6], leading to intra-abdominal inflammation, intestinal adhesion and ileus. When meconium peritonitis occurs, early operation is a valid way to prevent the exacerbation of intra-abdominal inflammation, hyperemia and edema of the intestinal wall and intestinal adhesion, assisting in the reduction of severe infection and mortality. Takino T et al. [13] pointed that the relative abundance of *Firmicutes* (including its families, such as *Lactobacillaceae*), which supports health and immune function, was increased substantially during the first week after birth. Giri P et al. [12] observed that the sympathoadrenal function could be affected by lack of a microbiome or microbial metabolites, leading to inferior health status. In our data, more than three fourths of patients in group A received surgery on the first day after birth, which means that they had more time for the bowel and intestinal microbiome to develop in the neonatal period. For patients in group B, after they were transferred to our department, examinations including chest radiography and echocardiography were required before anesthesia and surgery. All these necessary examinations were performed emergently but also depended on the schedule of the laboratory, radiography and ultrasound departments. It was believed that if the patients were born in our hospital, necessary examination before the surgery could be arranged in advance once their mothers were admitted to the delivery room due to the presence of abnormal gastrointestinal signs in the fetal period, which contributed to the early operation.

In addition, we analyzed hematological and biochemical indicators before the surgery to assess the general condition of these patients. Since the white blood cell count shows a variable range in the neonatal period, the CRP value and the results of ascitic culture were taken into account. We found that the positive rates of C-reaction protein evaluation and ascitic culture were higher in patients in group B than in patients from group A. Although the difference was not remarkable, such results reflected a severe inflammation in patients in group B and an increased risk of intra-abdominal infection due to delayed operation. In addition, bacterial infection of the bowel was more common in group B than in group A. As meconium is sterile, the existence of gut bacteria in ascites implies that new intestinal infection occurred after birth, partly reflecting the progression of MP. Hence, we hypothesize that surgery as early as possible for MP infants can avert the aggravation of edema and hyperemia of the intestinal wall and secondary infection.

In our cohort, the GA and BW of patients were higher in group A than in group B, suggesting that the general condition of group A patients was better. Patients in group B were transferred to our tertiary center from local hospitals, mainly rural hospitals, where the importance of routine prenatal examination and effective intervention measures are easily ignored. When gravidas were admitted to the obstetrics department in our center, clinical dietitians evaluated their nutritional status and planned a daily diet to maintain sufficient protein and calorie intake. During the monitoring of the fetus, if the dilation of bowel and ascitic fluid increased significantly and rapidly, after the fetus grew to maturity (more than 37 weeks), the obstetrician chose a time to deliver the infant to prevent further damage to the intestine of the fetus. If fetal distress or another emergency caused premature birth, dexamethasone was used for fetal lung maturation.

We presume that such prenatal treatments as reinforcement of nutrition for gravidas and monitoring of fetal growth made some contributions. Previous studies have shown that preterm birth and low birth weight are risk factors for MP patients [1, 6, 8–10]. However, there was no evidence to prove that the survival rate increased linearly with increasing GA and BW. Herein, we considered preterm birth and low birth weight as two classified variables. Statistically, the rates of preterm patients and low birth weight neonates were similar between the two groups. Additionally, cesarean section was conducted after the fetus was full-term and mature if dilatation of the bowel, ascites or intra-abdominal pseudocysts worsened along with gestational age in our center. No significant difference was observed in the survival rate between patients with different delivery modes in this study, although Wampach L et al. [14] noted that cesarean section could be detrimental to specific microbial strains and the immune-stimulatory potency of neonates which should be transmitted from mother by vaginally delivery. Therefore, further studies on the relationship among survival rate and gestational age, birth weight and delivery mode are needed.

For MP patients, the best surgical method is still controversial. In a previous study, Miyake H et al. [15] found that primary anastomosis requires a longer operation time but less time for parenteral nutrition and hospitalization for patients than enterostomy. As a result, primary anastomosis was considered in their department unless the patients, usually extremely low birth weight infants, could not tolerate long operative time or anesthesia. On the other hand, Nam S et al. [1] suggested that enterostomy was most appropriate for patients with massive intestinal adhesion or intensive edema of the intestinal mucosa. They emphasized that primary anastomosis, which requires less invasive procedures and avoids complications, such as loss of electrolytes and dermatitis around the stoma caused by enterostomy, could be preferred if the general condition of the patient is stable. In our center, the surgical procedure, mostly enterostomy and primary anastomosis, was decided according to the general condition, including the location of intestinal perforation, different diameters of these two ends of the proximal and distal bowel, extent of bowel wall edema and severity of intraabdominal inflammation and adhesion. Enterostomy, such as the double lumen or Bishop procedure, is an effective method to prevent anastomotic leakage and bowel obstruction, which could be caused by disorders of bowel motility. In this study, no significant difference was observed in the morbidity, survival rate and length of stay between these two kinds of procedures, with either parenteral or enteral nutrition.

Premature birth can cause several problems in neonates, such as low birth weight and immature organs in the neonatal period. Along with birth weight, the serum albumin value of neonates is closely related to gestational week of delivery [16]. Emil S et al. [17] mentioned that meconium ileus is more likely to occur in preterm infants than in full-term infants due to the low motility of the immature intestine. Research by Raslau D et al. [18] showed that low birth weight infants have an increased likelihood to have irritable bowel syndrome. A similar result was noticed in this study: among ten deaths, six occurred in preterm infants (three were preterm and low birth weight patients). By statistical analysis, not only birth weight and albumin level but also a reduction of approximately 20 cm of length of bowel after operation was recognized in preterm infants. Based on our experience, low birth weight and immature organs in preterm infants, especially the bowel and liver, will aggravate the situation and slow the recovery after the operation.

Moreover, Wang CN et al. [19] and Ping LM et al. [2] suggested that the formation of pseudocysts, which represent severe bowel injury, is another risk factor for MP patients. Nevertheless, the survival rate of 31 patients diagnosed as pseudocyst type by operation in our series was 90.3%, which was analogous to the study of Chan KW et.al. They mentioned that after meconium reaches the abdominal cavity from the perforation, the proximal intestine can be decompressed, and bowel dilation and intestinal wall edema can be lightened [20]. Additionally, the formation of pseudocysts can restrict the leakage of meconium and prevent infection spread. For MP patients of the pseudocyst type, removing the pseudocyst completely and performing peritoneal lavage in the operation were standard procedures in our treatment, which likely contributed to the high survival rate.

## Conclusions

According to our single-center study, early operation may be a critically important factor to improve the outcome of patients with meconium peritonitis. Prematurity and low birth weight are significant risk factors for MP. Prenatal management for fetuses with MP including monitoring of fetal growth, avoiding premature birth, reinforcement of nutrition for gravidas and delivery to tertiary hospitals, should be generalized, especially in developing countries.

## Data Availability

The datasets used and analysed during the current study are available from the corresponding author on reasonable request.

## Abbreviations

ALB: albumin
BW: Birth weight
CRP: C-Reaction protein
GA: Gestational age
LB: Low birth weight
LOS: Length of stay
M: Mean value
MP: Meconium peritonitis
MR: Magnetic resonance imaging
US: Ultrasound
